# Calibrating Deep Learning Classifiers for Patient-Independent Electroencephalogram Seizure Forecasting

**DOI:** 10.3390/s24092863

**Published:** 2024-04-30

**Authors:** Sina Shafiezadeh, Gian Marco Duma, Giovanni Mento, Alberto Danieli, Lisa Antoniazzi, Fiorella Del Popolo Cristaldi, Paolo Bonanni, Alberto Testolin

**Affiliations:** 1 Department of General Psychology, University of Padova, 35131 Padova, Italy; giovanni.mento@unipd.it (G.M.); fiorella.delpopolocristaldi@unipd.it (F.D.P.C.); 2 Epilepsy and Clinical Neurophysiology Unit, Scientific Institute, IRCCS E. Medea, 31015 Conegliano, Italy; gianmarco.duma@lanostrafamiglia.it (G.M.D.); alberto.danieli@lanostrafamiglia.it (A.D.); lisa.antoniazzi@lanostrafamiglia.it (L.A.); paolo.bonanni@lanostrafamiglia.it (P.B.); 3 Padova Neuroscience Center, University of Padova, 35131 Padova, Italy; 4 Department of Mathematics, University of Padova, 35131 Padova, Italy

**Keywords:** seizure prediction, epilepsy, electroencephalography, cross-validation, machine learning, signal processing, model validation, domain adaptation, model calibration, cross-subject

## Abstract

The recent scientific literature abounds in proposals of seizure forecasting methods that exploit machine learning to automatically analyze electroencephalogram (EEG) signals. Deep learning algorithms seem to achieve a particularly remarkable performance, suggesting that the implementation of clinical devices for seizure prediction might be within reach. However, most of the research evaluated the robustness of automatic forecasting methods through randomized cross-validation techniques, while clinical applications require much more stringent validation based on patient-independent testing. In this study, we show that automatic seizure forecasting can be performed, to some extent, even on independent patients who have never been seen during the training phase, thanks to the implementation of a simple calibration pipeline that can fine-tune deep learning models, even on a single epileptic event recorded from a new patient. We evaluate our calibration procedure using two datasets containing EEG signals recorded from a large cohort of epileptic subjects, demonstrating that the forecast accuracy of deep learning methods can increase on average by more than 20%, and that performance improves systematically in all independent patients. We further show that our calibration procedure works best for deep learning models, but can also be successfully applied to machine learning algorithms based on engineered signal features. Although our method still requires at least one epileptic event per patient to calibrate the forecasting model, we conclude that focusing on realistic validation methods allows to more reliably compare different machine learning approaches for seizure prediction, enabling the implementation of robust and effective forecasting systems that can be used in daily healthcare practice.

## 1. Introduction

Epilepsy is a chronic neurological disease characterized by repeated spontaneous interruptions in normal brain activity, often manifested as epileptic seizures [1]. Seizure attacks have a profound impact on various aspects of an individual’s life, including the physical, psychological, and social domains [2], and can have severe consequences, such as loss of consciousness or disruption of bladder function, leading to a significant reduction in quality of life [3]. Although more than 60% of the patients can control their seizures with medicines and another 10% can benefit from brain surgery, further advances in treatment are needed to improve the condition of epileptic people [4,5].

EEG is a valuable tool for the diagnosis of epilepsy due to its capability to capture anomalous electrical patterns in the brain with high temporal resolution at an affordable cost [6,7]. This non-invasive method is widely used to monitor the neuronal activity of the patient and detect epileptic discharges [8,9]. However, in addition to localizing and classifying seizures [10], forecasting epileptic activity before it occurs would be essential to reduce the consequences of attacks, for example, by giving patients and clinicians enough time to take the necessary action [11].

Despite decades of research on automatic seizure detection and forecasting [12,13,14], the latter task turns out to be extremely challenging [15]. Nevertheless, inspired by the successes of artificial intelligence (AI) in clinical diagnosis [16] and disease forecasting [17], consistent research efforts are being made to tackle the seizure prediction problem using advanced deep learning techniques [18,19,20]. For example, a study reported sensitivity rates of 96% and 94% in two different benchmark datasets [21], while another study reported an accuracy of almost 100% [22].

However, most published studies rely on the conventional use of randomized cross-validation (RCV) to assess model performance, while it has been argued that clinical applications of AI should be tested using more stringent validation methods [23]. The RCV method increases the risk of overfitting, because the training and test sets contain data from all patients; more robust evaluation procedures should test the forecasting model in a patient-independent way, for example, by using leave-one-patient-out (LOO) validation methods that completely exclude the data of the target patient from the training set [24,25,26]. Several studies have shown that achieving high forecast accuracy is very challenging under patient-independent conditions [27,28], but performance can be improved using domain adaptation techniques [29].

In this study, we address this problem by proposing an alternative framework based on patient-independent calibration. In particular, we ask whether the generalization of forecasting models can be significantly improved by fine-tuning the model on a few seizure events recorded from left-out (i.e., unseen) patients. To this end, we compare the performance of deep learning models for seizure forecasting under randomized and leave-out validation schemes, and for the latter, we investigate whether performance can be improved by exploiting a calibration method that relies on a single (Cal1) or a pair (Cal2) of seizures. We evaluate the proposed method using two different datasets, and compare deep networks against a standard machine learning approach. Compared to existing methods, our approach guarantees that the model’s accuracy is evaluated using independent data samples, which is a critical criterion to build forecasting methods that can be used in clinical practice.

The paper is structured as follows. In the first part, we explain the details of the datasets considered and their labeling procedure. After that, we describe the signal pre-processing pipeline, the deep learning model optimized for solving the forecasting task, and the metrics used to evaluate its performance. We then introduce our calibration method and report the experimental results. We conclude the article by discussing the limitations of our study and the most promising directions for future research.

## 2. Materials and Methods

### 2.1. EEG Datasets

We used two long-term continuous multichannel EEG datasets recorded at a sampling rate of 256 Hz and the international standard 10–20 scalp electrode positioning system. To ensure a sufficient distance from the ictal state and normal brain activity for the interictal state, only patients with at least one seizure with more than four hours of data prior to the seizure were selected [30]. Patients with a single seizure were only used to train the models, while patients with at least two seizures were eligible to study leave-out validation and calibration methods.

The first dataset was the popular CHB-MIT [31,32], in which we selected 22 common channels from 19 patients (15 men and 4 women), totaling 89 total seizures after removing patients chb12, chb13, chb15, chb23 and chb24 according to the selection criteria stated above. Eight out of these nineteen patients were eligible for validation and calibration. The second dataset, which we call Conegliano throughout this paper, contained 20 common channels of 22 patients (10 men and 12 women) with a total of 77 seizures, recorded by the Epilepsy and Clinical Neurophysiology Unit of Eugenio Medea IRCCS Hospital in Conegliano, Italy, during a standard clinical protocol of continuous patient monitoring. Eight out of twenty-two patients in the Congeliano dataset were eligible for validation and calibration.

### 2.2. Data Labeling

In the forecasting of epileptic EEG signals, two states before a seizure were considered: preictal signals coming before a seizure, and normal interictal brain activity occurring far from a seizure [33]. Since there is no standard to define the duration of a preictal state, different periods ranging from 10 to 90 min are generally considered [34]. In this study, after exploring various configurations between 10 and 40 min, we decided to select 15 min before a seizure as the target preictal state, since this configuration allowed to generate enough training data from each patient while preserving the distinctiveness of preictal states from normal brain activity. The beginning and end of the ictal state of the CHB-MIT dataset were extracted from the official website, while the Conegliano dataset was manually marked by two clinicians based on video-EEG monitoring information.

**Figure 1:**
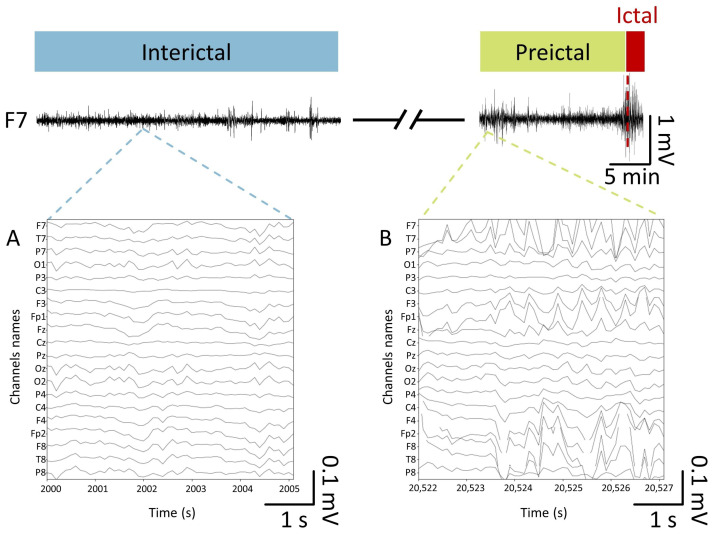
Segmentation of the pre- and interictal states for the binary seizure forecasting task. The trace depicts 45 min of an EEG recording from the F7 channel of the Conegliano dataset during a seizure. Panels (**A**,**B**) illustrate a magnification of 5 s of recordings from 20 common channels of the inter- and preictal states, respectively.

After applying a four-hour interval between the preictal and interictal states, we selected up to 60 min of data for the interictal class to reduce the probability of encountering abnormal brain activity related to the preictal state [35]. Figure 1 represents our schematic signal labeling process to distinguish between preictal and interictal states, including two images of recordings from epileptic patients from the Conegliano dataset.

### 2.3. EEG Signal Pre-Processing

The signal was pre-processed by applying notch filters at 50 and 100 Hz to mitigate power line interference [36], a high-pass filter at 1 Hz to remove DC offset and baseline fluctuations [37,38], and a low-pass filter at 125 Hz to maintain higher frequencies that could characterize abnormal brain activity [39,40]. Both datasets were also downsampled to 128 Hz to reduce the computational cost of model training [41,42]. EEG signals were divided into time windows before being given as input to the deep learning models. We explored different window sizes (1, 5, 10, and 30 s) to establish the most effective input format, which turned out to be 5 s. Data pre-processing was implemented using Python (version 3.8.5) and the MNE package [43].

To balance the binary classification task, we undersampled the number of data samples in the interictal state by randomly selecting 15 min of contiguous data [44]. In the RCV setting, the signal was standardized by computing the average and standard deviation of the training set after splitting. In the LOO setting, instead, each training patient was standardized separately, while test patients were standardized using the average and std of all training patients to avoid information leakage [45]. In the calibration procedure, we used the average and std of the calibration data (one or two seizures) to standardize the entire signal of the target test patient.

### 2.4. Deep Learning Model

Seizure forecasting was carried out using a convolutional neural network (CNN), which was implemented using the PyTorch framework (version 1.13.0) [46] and trained on a virtual machine equipped with an NVIDIA V100 GPU allocated on the Google Cloud Platform.

Although we had a two-dimensional input shape (number of common channels × time window), the kernels moved in one direction in the early convolutional layers and then in two directions in the subsequent convolutional layers. This approach was adopted to better exploit the information on interchannel correlations between EEG channels [47,48]. The model architecture and learning hyperparameters were optimized using a hierarchical strategy (see [48] for details), which considered the number of hidden layers (3 to 7), number of kernels (8, 16, 32, and 64), kernel size (2, 3, 5, and 7), pooling size (2, 3, and 5), number of dense layers (1 to 4), number of dense units (32, 64, 128, and 256), number of dropout layers (1 to 8), dropout rate (0.1, 0.2, and 0.5), learning rate (0.01, 0.005, 0.001, 0.0002 and 0.0001), and batch size (16, 32, 64, 128). Learning was performed using the Adam optimizer [49] with binary cross-entropy loss, using an early-stopping criterion.

**Figure 2:**
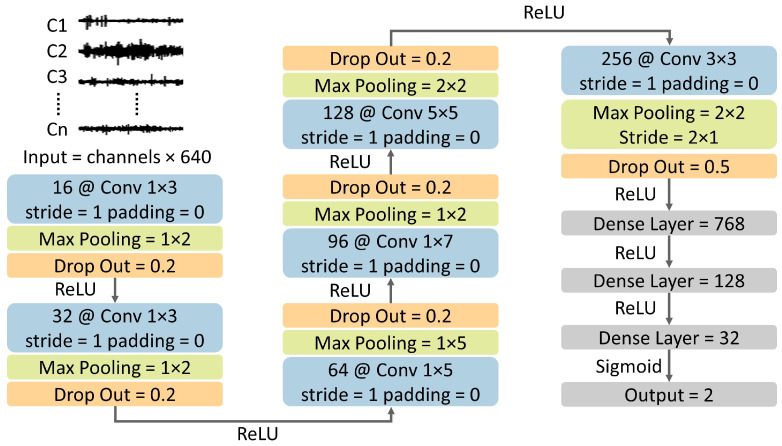
The deep learning architecture contains six convolutional layers followed by batch normalization, pooling, and drop-out layers. Three dense layers are finally used to produce the output prediction.

The final architecture consisted of six CNN layers with batch normalization and Rectified Linear Units (ReLU) (see Figure 2 for a schematic representation). The stride of the kernels in all layers was 1 × 1 (no padding), and the number and shape of these kernels were 16@1 × 3, 32@1 × 3, 64@1 × 5, 96@1 × 7, 128@5 × 5, and 256@3 × 3, respectively. The max-pooling layers after each CNN layer were of size 1 × 2, 1 × 2, 1 × 5, 1 × 2, 2 × 2, and 2 × 2, respectively. Six dropout layers were placed after each pooling layer, with a drop rate of 0.2, except for the last dropout layer, which had a rate of 0.5. After flattening each data point into 768 nodes, two dense layers with 128 and 32 hidden units were applied. A sigmoid unit finally produced the binary classification output, encoding the discrimination between pre- and interictal states.

### 2.5. Model Evaluation

We benchmarked our deep learning model against an Extreme Gradient Boosting (XGBoost), a standard machine learning classifier that we trained on a set of 53 features extracted from the EEG signal (for details, see [26]). The models were evaluated by computing the true positive (*tp*), false positive (*fp*), true negative (*tn*) and false negative (*fn*) rates on the test set. These indicators were used to calculate accuracy (ACC), sensitivity (SEN), and specificity (SPE), which are the standard metrics used to evaluate machine learning algorithms for seizure forecasting [50]:

(1) $$\begin{array}{cc} \; & ACC=((tp+tn)/(tp+tn+fn+fp)),\\ \; & SEN=(tp/(tp+fn)),\\ \; & SPE=(tn/(tn+fp)). \end{array}$$

Accuracy is simply defined as the percentage of correct (true positives or true negatives) responses over the entire set of test observations. Despite its intuitive meaning, accuracy is not representative of model performance in presence of unbalanced data, which is often the case in medical diagnosis. Sensitivity (also known as true positive rate) is the probability of a positive test result, conditioned on the individual truly being positive. This metric allows to refine the clinical evaluation, since a highly sensitive test implies that there are few false negative results, and thus fewer cases of disease (seizure events, in our case) are missed. Specificity (also known as true negative rate) instead represents the probability of a negative test result, conditioned on the individual truly being negative. This metric complements the information provided by Sensitivity, since a highly specific test implies that there are a few false positive results.

We evaluated the models using both a RCV scheme, implemented through a five-fold cross-validation considering all patient data, and a LOO scheme, where one targeted patient data were ultimately considered as the test set, while the rest of the patients were included in the training set. Since achieving high accuracy in the LOO setting is extremely challenging, we considered this validation scheme as the baseline to evaluate the performance gain of the proposed calibration method.

### 2.6. Calibration Method

**Figure 3:**
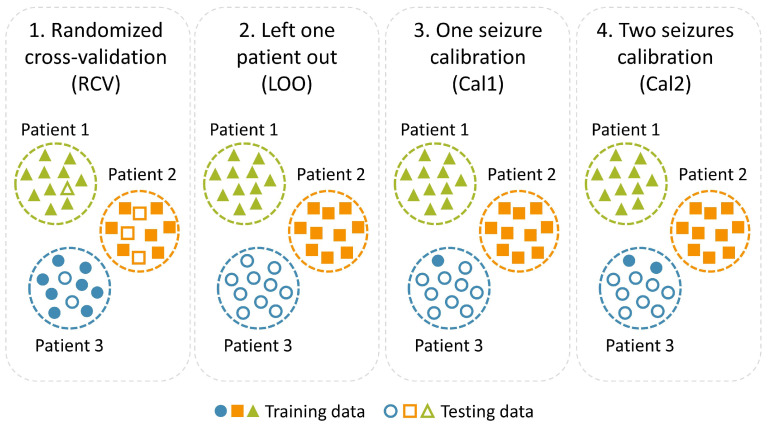
Graphical representation of the two validation settings (RCV and LOO) considered in our experiments and the proposed calibration method, which exploits just one (Cal1) or two (Cal2) seizures of the target patient to fine-tune the forecasting model.

The proposed calibration method is illustrated in Figure 3. We postulated that accuracy in the LOO setting could be improved by fine-tuning the model using one or more seizure events recorded from the left-out patient under investigation: in the Cal1 version, we exploited a single seizure to calibrate the model, thus including in the training set one epileptic event featuring at least four hours of pre-seizure recording from the target patient. At the end of the training phase, the model was tested with the remaining data of the target patient. In the Cal2 version, we included two seizures of the target patient in the training set. The first seizure was the same one used in Cal1, while the second was randomly selected from the rest of the seizures available for that patient. In patients with only one seizure with more than four hours of preceding data, the interictal state of the first seizure was considered normal brain activity for the second seizure to balance the calibration data points.

It should be noted that model fine-tuning in Cal1 and Cal2 was carried out starting from the CNN configuration obtained in the LOO baseline. We believe that such a two-stage training procedure is more realistic than a single-stage training procedure, where the CNN is simply trained from scratch on all training data, since in clinical settings the goal should be to quickly adapt a pre-trained model (LOO baseline) with patient-specific seizure data, rather than training a new CNN model on all available data.

The calibration phase of deep learning models can be carried out very efficiently: in our specific case, the fine-tuning calibration phase required between 5 min and 10 min to complete, which we believe could be considered a reasonable time for deployment in real-world clinical settings.

## 3. Results

### 3.1. CHB-MIT Dataset

**Figure 4:**
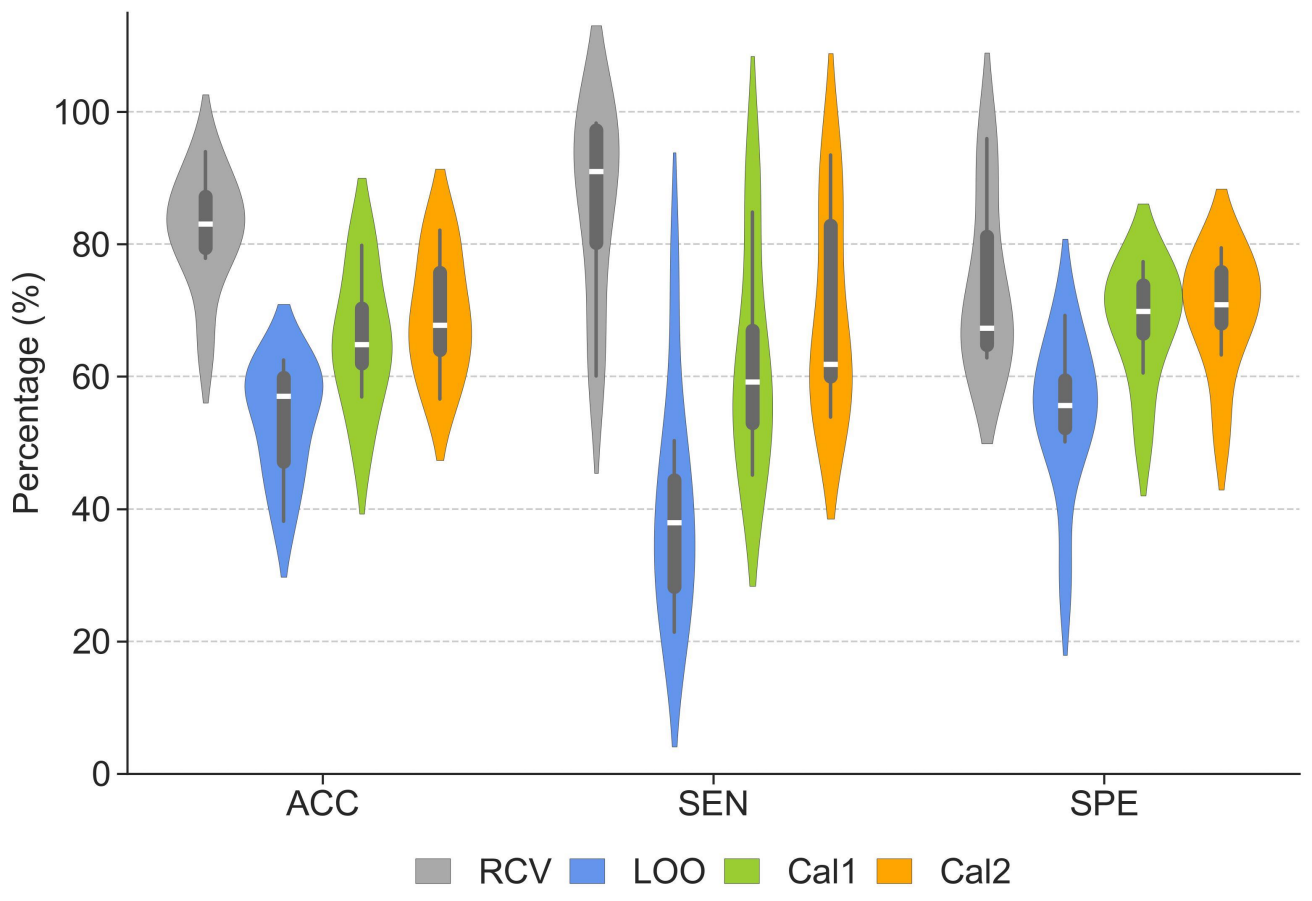
Performance of the CNN model in the CHB-MIT dataset obtained with randomized cross-validation (RCV), leave-one-patient-out (LOO) validation and after Cal1 and Cal2 calibration. The violin plots illustrate the distribution of ACC, SEN, and SPE. The box plots with horizontal lines represent the interquartile range and the median.

The performance obtained in the CHB-MIT dataset is reported in Figure 4. As expected, the results show that RCV can lead to very high performance in terms of all evaluation metrics, but these numbers dramatically drop when the model is tested under the more realistic LOO validation condition.

Nevertheless, the performance significantly improves following model calibration. Even using one single seizure from the left-out patient allows us to increase ACC, SEN, and SPE of 12%, 22%, and 14%, respectively, compared to the LOO baseline. Introducing a second seizure for calibration allows us to further improve the forecast performance, leading to an increase of 16%, 29%, and 16% compared to the baseline. Detailed evaluation metrics for each patient are reported in Table 1, along with information about gender and number of available seizures. A statistical comparison was applied to the LOO, Cal1, and Cal2 performance metrics to evaluate the improvement over the baseline resulting from two calibration approaches. The results of repeated measures analysis of variance (ANOVA) reported in Table 2 show significant differences (*p*-value < 0.001) in ACC, SEN, and SPE.

Although epileptic patients have similar symptoms, their underlying brain dynamics might be quite heterogeneous due to the different causes of epilepsy. Although this might lead to an increase in variability in forecasting performance between patients, we can still observe some consistent trends in our results. For example, patient chb22 obtains the best accuracy among all patients in the RCV condition (93.99%) and, despite this number falling below the average accuracy in the LOO condition, it improves again to the best score after calibrating with just one seizure (79.84%). This suggests that the RCV performance was likely biased by overfitting, and that our calibration method can significantly mitigate this phenomenon in the LOO case. In the case of patient chb10, after calibration with two seizures, the accuracy is comparable to that achieved in the RCV setup, and for patient chb09, the values of accuracy and sensitivity after calibration with two seizures are remarkably high (ACC of 82.11% and SEN of 93.47%), demonstrating that our calibration method is a promising solution to improve forecast accuracy in the challenging LOO condition.

**Figure 5:**
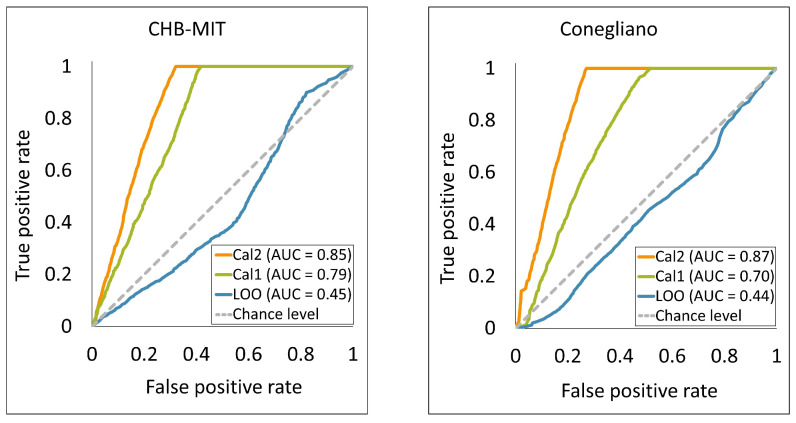
The receiver operating characteristic (ROC) curves and the area under the curve (AUC) for LOO, Cal1, and Cal2 methods in the CHB-MIT and Conegliano datasets. y-axis and x-axis correspond to the true positive rate (sensitivity) and false positive rate (1—specificity), respectively.

The receiver operating characteristic (ROC) curves for LOO, Cal1, and Cal2 are illustrated in the left panel of Figure 5, allowing for a more systematic comparison between LOO and the performance of the calibration methods. Notably, the area under the curve (AUC) for the calibration methods increased by approximately 0.34 and 0.40, respectively.

### 3.2. Conegliano Dataset

**Figure 6:**
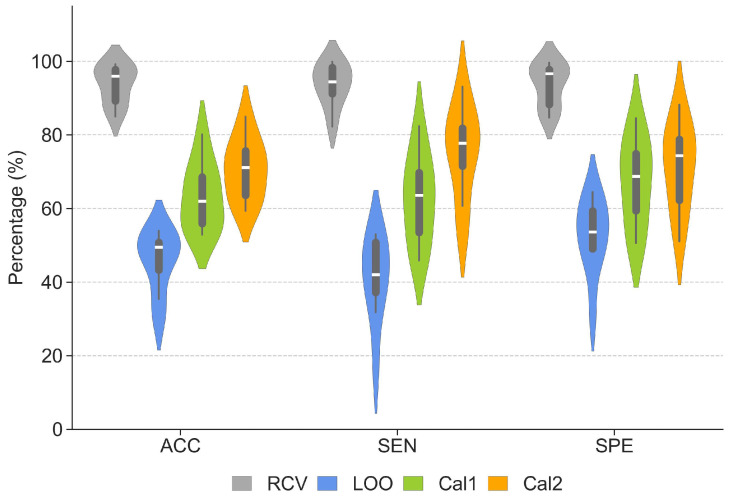
Performance of the CNN model in the Conegliano dataset obtained with randomized cross-validation (RCV), leave-one-patient-out (LOO) validation and after Cal1 and Cal2 calibration. The violin plots illustrate the distribution of ACC, SEN, and SPE. The box plots with horizontal lines represent the interquartile range and the median.

The results obtained in the Conegliano dataset are reported in Figure 6. As observed with the CHB-MIT dataset, randomized cross-validation seemingly leads to impressive performance, but all evaluation metrics dramatically drop when the model is tested under the more realistic LOO condition.

Nevertheless, also with the Conegliano dataset after model calibration we obtain significant improvements in all metrics, with ACC, SEN and SPE gains of 15%, 10% and 16% for Cal1 and 23%, 22% and 30% for Cal2. The ROC curves of LOO, Cal1, and Cal2 for the Conegliano dataset are illustrated in the right panel of Figure 5. The AUC improved by approximately 0.26 and 0.43 for Cal1 and Cal2, respectively. Detailed evaluation metrics for each patient are reported in Table 3. The performance of the two calibration versions was evaluated by applying a statistical comparison to the LOO, Cal1, and Cal2 performance metrics. Repeated ANOVA tests demonstrated significant differences (*p*-value < 0.001) between the calibration methods and the baseline in ACC, SEN, and SPE. The average and std of the different methods and the results of the statistical tests are described separately in Table 4.

Also in this case, we observe promising results with several patients, pointing to the generalization of the proposed calibration method. For example, after calibration with two seizures, p4, p5, and p6, achieve an ACC of 75.13%, 84.96%, and 77.23%, respectively. Furthermore, p1 and p6, which obtained a very poor accuracy in the LOO condition, improved by 23.96% and 47.30%, respectively, demonstrating that the proposed calibration method can lead to impressive performance gains even in patients with low baseline performance.

### 3.3. How Many Seizures for Calibration?

The results presented in Figure 4, Figure 5 and Figure 6 indicates that the use of two seizures rather than one to calibrate the model could lead to a further increase in performance in both datasets. However, Tukey post hoc analysis did not show statistical differences between the two calibration versions in either CHB-MIT or Conegliano (for details about the statistical results, see Table 5); therefore, our current results do not allow us to establish a statistical difference between these two variants of the calibration method.

**Figure 7:**
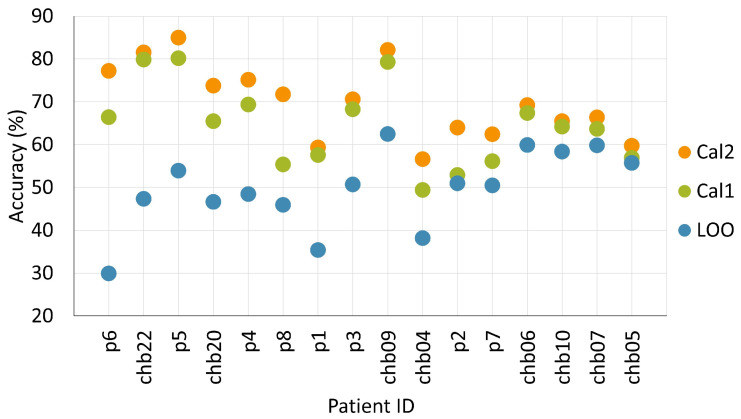
Comparison of accuracy gains obtained by Cal1 and Cal2 with respect to the LOO baseline across all patients. Patients are sorted according to the maximum gain obtained by Cal2.

Nevertheless, differences might emerge by expanding the sample size, and the overall trends suggest that using more seizures is more effective in fine-tuning the CNN model. This intuition is confirmed by the data reported in Figure 7, which shows the accuracy gains obtained by the two calibration versions across all patients in the two datasets, ordered according to the maximum gain achieved by Cal2 with respect to the LOO baseline. The plot clearly shows that using two seizures for calibration (Cal2) always leads to an increase in accuracy compared to using a single seizure (Cal1), suggesting that calibration could benefit from a prolonged tuning phase on the target patient.

### 3.4. Comparison with a Standard Machine Learning Classifier

We finally investigated whether our calibration method could also be used with other machine learning algorithms, comparing the gains obtained by the CNN against those obtained by a more standard supervised machine learning model implemented as an XGBoost classifier [51] and trained on a set of standard features extracted from the EEG recordings. These features contained time-domain features such as mean, variance, standard deviation, skewness, and kurtosis, and essential frequency-domain features such as power spectral density, spectral entropy, and Hjorth parameters (for details, see [26]).

**Figure 8:**
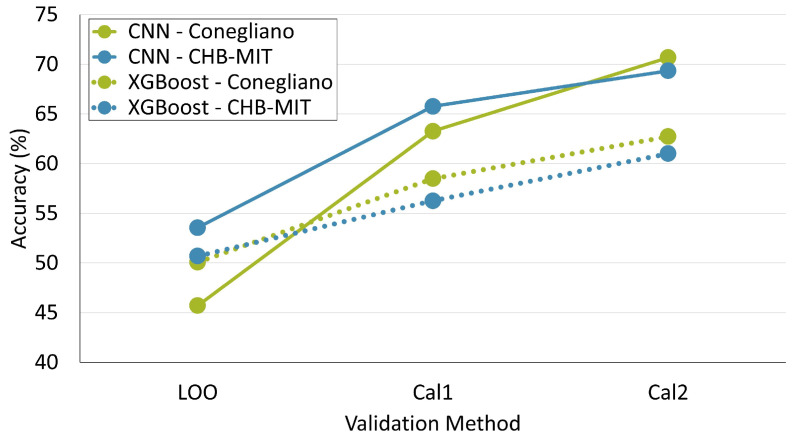
Comparison between the CNN model (solid lines) and the XGBoost classifier (dotted lines) in terms of accuracy gain for the two calibration versions with respect to the LOO baseline. The blue lines refer to the CHB-MIT dataset while the green lines refer to the Conegliano dataset.

It turns out that our calibration method is also effective with XGBoost, although the performance gains are slightly lower compared to the CNN. The improvement in accuracy resulting from the use of one and two calibration seizures is shown in Figure 8, while the detailed evaluation metrics are reported in Table 6. In the CHB-MIT dataset, the ACC of the XGBoost classifier improved by 6% (Cal1) and 10% (Cal2), while in the Conegliano dataset, it improved by 8% (Cal1) and 13% (Cal2).

## 4. Discussion

In this study, we investigated the performance of automatic seizure forecasting algorithms using two datasets of raw multichannel EEG recordings. We focused on deep learning models, implementing a convolutional neural network (CNN) architecture that was optimized to accurately distinguish between interictal and preictal brain states. We compared the performance of the CNN model obtained in the most commonly used randomized cross-validation (RCV) condition with that obtained in a more challenging, but realistic, leave-one-patient-out (LOO) condition.

As expected, the RCV resulted in a very high forecast accuracy. In particular, the deep learning model introduced in this work outperformed previous results obtained with the same datasets using more traditional machine learning pipelines [26], according to all evaluation metrics. However, performance in left-out patients decreased dramatically. At the same time, we showed that fine-tuning the LOO model using one or two seizures from left-out patients can significantly improve LOO performance in terms of all evaluation metrics: accuracy, sensitivity, and specificity. This is particularly relevant in clinical settings, where the goal is to improve accuracy but also to ensure that the forecasting model produces few false negatives and few false positives.

Improvement in performance was observed in both datasets and, although the specific gains were heterogeneous, calibration led to an increase in accuracy for all patients. Furthermore, increasing the number of calibration seizures further boosted performance, achieving an up to 25% accuracy gain in a CHB-MIT patient and up to 47% in a Conegliano patient. It thus seems that, in general, it might be preferable to use the Cal2 method (or even further increasing the number of calibrating events), although it should be noted that being able to calibrate a forecasting system with minimal amount of data, as with Cal1, could be desirable in situations of data scarcity. Our findings also demonstrated that the proposed calibration method could be used with standard machine learning algorithms, although performance gains were more marked with deep neural networks.

Table 7 compares our results with those obtained in other recent studies that proposed to apply adaptation methods to improve the performance of machine learning classifiers in the CHB-MIT dataset under cross-subject conditions. Although such a comparison should be treated with caution, since these studies exploited different validation procedures to test the model performance, it still indicates that our results are consistent with those reported in previous work. It should also be noted that some of these approaches require to train multiple models for each seizure [52,53], which increases the computational burden and might be extremely time consuming in the case of large datasets.

## 5. Conclusions

The primary objective of this work was to demonstrate that by introducing calibration procedures we can significantly improve automatic seizure forecasting algorithms even in challenging leave-patient-out settings. Indeed, although many studies have reported high performance with patient-specific approaches, building a clinical forecasting system requires to develop patient-independent approaches that could be used in new epileptic subjects with minimal tuning.

The proposed calibration method is easy to implement and guarantees a significant improvement in forecasting performance, even with the use of a single calibrating seizure. We believe that this constitutes an important first step to enable the implementation of forecasting devices that could be finally used in clinical practice. For example, clinicians might initially develop and deploy a generic forecasting model, which is then personalized on each individual patient after the recording of one (or a few) epileptic events. However, at the same time, it should be noted that the proposed calibration method requires to have at least one seizure recorded in each independent patient, which constitutes a serious limitation of our approach since it prevents its use with individuals who have never had a seizure but are still considered at high risk. Future research should thus design calibration procedures that can be used with individuals without prior seizures, for example by exploiting EEG signals recorded during normal daily activity or by using additional information, such as biomarkers or data extracted from clinical records.

Furthermore, the non-negligible variability of forecasting accuracy between patients suggests that further efforts should be spent to improve the reliability of predictive models. The sources of variation can be extremely heterogeneous [56,57] and likely depend on the etiology of the seizure, its spatial source (e.g., temporal lobe, hippocampus, parietal lobe, etc.), the age and overall health condition of the patient, the severity of the epilepsy, the time from the first appearance of the epileptic condition, the details of the EEG registration devices (e.g., sensor cap) and possibly many other factors. Augmenting the models by adding other variables as potential predictors might therefore be a promising research direction to further boost the performance of forecasting systems and make them more tailored for each patient.

In conclusion, building a generalized seizure forecasting system remains an extremely challenging task, given the considerable variability between epileptic patients [58,59] and the variability of seizure events even within the same patient [60]. More research is still needed to establish a reliable forecasting system that could finally be used in the routine health care of people with epilepsy.

## Figures and Tables

**Table 1:** Performance of the CNN model for each patient in the CHB-MIT dataset. Each row corresponds to the ID, gender, and number of seizures per patient followed by ACC, SEN, and SPE values. Maximum values are highlighted in bold.

			RCV			LOO			Cal1			Cal2		
ID	Gend.	No. Seizures	ACC (%)	SEN (%)	SPE (%)	ACC (%)	SEN (%)	SPE (%)	ACC (%)	SEN (%)	SPE (%)	ACC (%)	SEN (%)	SPE (%)
chb04	m	3	84.11	66.86	**95.98**	38.16	27.03	56.24	49.40	45.12	60.56	56.59	53.84	63.24
chb05	f	5	79.94	85.71	62.80	55.71	33.60	50.10	56.92	45.43	68.47	59.72	55.78	69.58
chb06	f	7	82.05	60.11	91.47	59.90	42.33	63.36	67.35	58.95	76.37	69.22	62.30	78.41
chb07	f	3	77.86	97.31	64.71	59.83	50.35	55.05	63.66	59.47	**77.37**	66.36	61.49	**79.46**
chb09	f	3	87.74	96.27	65.45	**62.47**	**76.54**	29.48	79.28	**91.61**	50.72	**82.11**	**93.47**	51.77
chb10	m	7	64.63	97.17	64.78	58.37	42.37	52.91	64.21	55.52	68.99	65.49	61.47	70.66
chb20	f	6	87.03	84.66	77.74	46.66	28.66	58.15	65.49	61.01	70.71	73.75	80.87	71.14
chb22	f	3	**93.99**	**98.31**	69.22	47.33	21.36	**69.20**	**79.84**	84.87	72.96	81.55	88.66	74.95
		Average	82.17	85.80	74.02	53.55	40.28	54.31	65.77	62.75	68.27	69.35	69.74	69.90

**Table 2:** The average ACC, SEN, and SPE of the CNN model obtained from LOO, Cal1, and Cal2 in the CHB-MIT dataset, represented by the mean (%) ± std. The last two columns report the F-value and *p*-value from the ANOVA test.

Metrics	LOO (Mean ± Std)	Cal1 (Mean ± Std)	Cal2 (Mean ± Std)	F-Value	*p*-Value
ACC	53.55 ± 8.54	65.77 ± 10.26	69.35 ± 9.34	14.30	<0.001
SEN	40.28 ± 17.47	62.75 ± 16.96	69.74 ± 15.51	15.24	<0.001
SPE	54.31 ± 11.70	68.27 ± 8.82	69.90 ± 8.98	36.79	<0.001

**Table 3:** Performance of the CNN model for each patient in the Conegliano dataset. Each row corresponds to the ID, gender, and number of seizures per patient followed by ACC, SEN, and SPE values. Maximum values are highlighted in bold.

			RCV			LOO			Cal1			Cal2		
ID	Gend.	No. Seizures	ACC (%)	SEN (%)	SPE (%)	ACC (%)	SEN (%)	SPE (%)	ACC (%)	SEN (%)	SPE (%)	ACC (%)	SEN (%)	SPE (%)
p1	m	4	97.12	98.00	96.09	35.40	38.96	31.46	57.58	68.72	50.55	59.36	76.69	51.10
p2	m	5	89.33	93.18	84.70	50.97	43.23	60.44	52.88	49.33	61.93	63.96	83.61	62.46
p3	f	5	88.82	88.82	88.83	50.71	**52.97**	48.73	68.24	54.87	**84.53**	70.61	60.68	**88.21**
p4	f	6	94.84	91.98	97.66	48.45	31.80	**64.46**	69.35	62.28	74.17	75.13	78.76	74.68
p5	f	4	**99.18**	**99.86**	97.93	**53.91**	50.63	58.98	**80.15**	**82.35**	77.02	**84.96**	**93.10**	79.54
p6	f	3	98.48	99.10	97.20	29.93	16.35	57.46	66.42	72.71	64.51	77.23	75.14	78.45
p7	f	7	97.46	95.68	**99.60**	50.47	51.04	49.80	56.11	64.82	51.89	62.41	81.19	61.74
p8	m	10	84.98	82.27	86.61	45.94	40.80	49.12	55.39	45.90	72.93	71.72	53.79	74.05
		Average	93.78	93.61	93.58	45.72	40.72	52.56	63.27	62.62	67.19	70.67	75.37	71.28

**Table 4:** The average ACC, SEN, and SPE of the CNN model obtained from LOO, Cal1, and Cal2 in the Conegliano dataset, represented by the mean (%) ± std. The last two columns report the F-value and *p*-value from the ANOVA test.

Metrics	LOO (Mean ± Std)	Cal1 (Mean ± Std)	Cal2 (Mean ± Std)	F-Value	*p*-Value
ACC	45.72 ± 8.50	63.27 ± 9.34	70.67 ± 8.53	28.00	<0.001
SEN	40.72 ± 12.18	62.62 ± 12.24	75.37 ± 12.60	19.97	<0.001
SPE	52.56 ± 10.34	67.19 ± 12.10	71.28 ± 11.96	16.03	<0.001

**Table 5:** Tukey post hoc tests comparing the performance of calibrated and baseline models in the CHB-MIT and Conegliano datasets. Each row reports the *p*-value resulting from the comparison of ACC, SEN, and SPE metrics.

Dataset	Validation Methods	ACC	SEN	SPE
	LOO—Cal1	<0.05	<0.05	<0.05
CHB-MIT	LOO—Cal2	<0.01	<0.01	<0.05
	Cal1—Cal2	0.73	0.68	0.94
	LOO—Cal1	<0.01	<0.01	<0.05
Conegliano	LOO—Cal2	<0.01	<0.01	<0.05
	Cal1—Cal2	0.23	0.12	0.76

**Table 6:** Average ACC, SEN, and SPE obtained by the XGBoost classifier in the CHB-MIT and Conegliano datasets (mean (%) ± std).

Dataset	Metrics	LOO	Cal1	Cal2
	ACC	50.70 ± 7.83	56.26 ± 4.40	61.02 ± 5.81
CHB-MIT	SEN	44.02 ± 10.38	52.89 ± 11.66	58.44 ± 10.47
	SPE	60.95 ± 13.34	63.44 ± 12.65	66.84 ± 13.13
	ACC	50.08 ± 6.71	58.49 ± 5.70	62.73 ± 4.71
Conegliano	SEN	46.06 ± 19.54	69.22 ± 17.07	73.52 ± 16.34
	SPE	50.21 ± 13.02	65.85 ± 11.44	70.68 ± 14.66

**Table 7:** A comparison of different studies exploiting domain adaptation methods for cross-subject seizure forecasting in the CHB-MIT dataset.

Authors	Year	Input Type	Classifier	SEN (%)	AUC
Peng et al. [29]	2022	spectrograms	Autoencoder	73	-
Zhao et al. [54]	2023	raw signal	Gaussian mixture	71	0.68
Liang et al. [52]	2023	raw signal	CNN	89	0.85
Zhang et al. [53]	2023	spectrograms	Transformer	80	0.81
Jemal et al. [55]	2024	raw signal	CNN	-	0.75
**This work**	2024	raw signal	CNN	70	0.85

## Data Availability

The data used in the present study is not publicly available due to privacy issues related to the involvement of clinical populations.

## Abbreviations

The following abbreviations are used in this manuscript:

EEG	Electroencephalography
AI	artificial intelligence
RCV	Randomized cross-validation
LOO	Leave-one-patient-out
Cal1	Calibration with one seizure
Cal2	Calibration with two seizures
CNN	Convolutional Neural Network
XGBoost	Extreme Gradient Boosting
ReLU	Rectified Linear Unit activation function
tp	true positive
fp	false positive
tn	true negative
fn	false negative
ACC	Accuracy
SEN	Sensitivity
SPE	Specificity
ANOVA	Analysis of variance
ROC	receiver operating characteristic
AUC	area under the curve
